# Analysis of the distal urinary tract in larval and adult zebrafish reveals homology to the human system

**DOI:** 10.1242/dmm.050110

**Published:** 2023-07-19

**Authors:** Ibrahim Jubber, Duncan R. Morhardt, Jonathan Griffin, Marcus G. Cumberbatch, Maggie Glover, Yang Zheng, Ishtiaq Rehman, Catherine A. Loynes, Syed A. Hussain, Stephen A. Renshaw, Steven D. Leach, Vincent T. Cunliffe, James W. F. Catto

**Affiliations:** ^1^ Department of Oncology and Human Metabolism, University of Sheffield, Sheffield S10 2RX, UK; ^2^ Department of Urology, Sheffield Teaching Hospitals NHS Foundation Trust, Sheffield S10 2JF, UK; ^3^ Department of Molecular and Systems Biology, Dartmouth Cancer Center, Geisel School of Medicine at Dartmouth, Hanover, NH 03755, USA; ^4^ Department of Histopathology, Sheffield Teaching Hospitals NHS Foundation Trust, Sheffield S10 2JF, UK; ^5^ Healthy Lifespan and Neuroscience Institute, Department of Biosciences, University of Sheffield, Sheffield S10 2TN, UK; ^6^ The Bateson Centre and Department of Infection, Immunity and Cardiovascular Disease, University of Sheffield, Sheffield S10 2TN, UK; ^7^ Department of Medical Oncology, Weston Park Hospital, Sheffield Teaching Hospitals NHS Foundation Trust, Sheffield S10 2SJ, UK; ^8^ School of Biosciences, University of Sheffield, Firth Court, Western Bank, Sheffield S10 2TN, UK

**Keywords:** Zebrafish, Urinary tract, Urinary bladder, Urethra, Uroplakins, Tetraspanins

## Abstract

Little is known about the distal excretory component of the urinary tract in *Danio rerio* (zebrafish). This component is affected by many human diseases and disorders of development. Here, we have undertaken multi-level analyses to determine the structure and composition of the distal urinary tract in the zebrafish. *In silico* searches identified *uroplakin 1a* (*ukp1a*), *uroplakin 2* (*upk2*) and *uroplakin 3*b (*upk3b*) genes in the zebrafish genome (orthologues to genes that encode urothelium-specific proteins in humans). *In situ* hybridization demonstrated *ukp1a* expression in the zebrafish pronephros and cloaca from 96 h post-fertilization. Haematoxylin and Eosin staining of adult zebrafish demonstrated two mesonephric ducts uniting into a urinary bladder that leads to a distinct urethral opening. Immunohistochemistry identified Uroplakin 1a, Uroplakin 2 and GATA3 expression in zebrafish urinary bladder cell layers that match human urothelial expression. Fluorescent dye injections demonstrated zebrafish urinary bladder function, including urine storage and intermittent micturition, and a urethral orifice separate from the larger anal canal and rectum. Our findings reveal homology between the urinary tracts of zebrafish and humans, and offer the former as a model system to study disease.

## Teaser

**Summary:** This study demonstrates close homology in urinary tract anatomy between zebrafish and human, including the presence of a distinct adult urinary bladder capable of periodic micturition.

## INTRODUCTION

The urinary tract is essential for the regulation of extracellular fluid and the excretion of metabolites. In humans, the urinary tract is composed of a multifunctional kidney, with excretory, metabolic and endocrine roles, and an excretory component. The excretory component comprises two propulsive drainage tubes (ureters) leading to a contractile expansive storage vesicle (urinary bladder) with intermittent outflow via the urethra. Micturition (passing urine) occurs under voluntary and involuntary neurological control. The embryological development of the urinary tract is characterized by complex interactions between various tissues.

In humans, the distal excretory component of the urinary tract begins development at ∼4 weeks gestation. The cloaca is partitioned by the urorectal septum to form the ventral urogenital sinus and the dorsal anorectal canal. The urogenital sinus develops into the urinary bladder and urethra. The mesonephric ducts fuse with the urogenital sinus and the ureteric bud develops as an outgrowth from the distal mesonephric duct. The ureteric bud is involved in reciprocal induction events, with the metanephric mesenchyme giving rise to the collecting ducts, renal pelvis and ureters. The distal mesonephric duct and ureteric bud undergo a remodelling process whereby the ureters fuse with the urogenital sinus, and subsequently migrate superiorly and laterally to form the vesicoureteric junctions and the trigone. The distal mesonephric ducts migrate inferiorly to empty into prostatic urethra and form the ejaculatory duct (Liaw et al., 2018).

The resultant excretory system is lined with urothelium – a multi-layered epithelium with a terminally differentiated superficial layer – as well as intermediate and basal cell compartments (Dalghi et al., 2020). The superficial layer is composed of ‘umbrella cells’, which are characterized by the expression of uroplakin proteins. Uroplakin proteins are from a superfamily of tetraspanins and are highly evolutionarily conserved across species (Garcia-Espana et al., 2006). They form an asymmetric unit membrane on the apical surface of the superficial urothelial layer. Uroplakin proteins have been reported to have barrier function with respect to water permeability (Hu et al., 2000, 2002). There are four important uroplakins in humans: 1a, 1b, 2 and 3. Basal cell layers express transcription factor tumour protein 63 (TP63 or p63) (Steurer et al., 2021) and high molecular weight cytokeratins, such as cytokeratin 5 (KRT5) (Volkel et al., 2022).

*Danio rerio* (zebrafish) models have been used to study the biology of the kidney. The zebrafish embryonic and larval kidney (pronephros) comprises two crescent-shaped nephrons fused in the midline that run from the glomerulus to the pronephric ducts and cloaca (Naylor and Davidson, 2017). The mesonephros (the permanent adult kidney structure in zebrafish) develops at around 10 days post-fertilization (dpf), during the transition from larva to juvenile as the osmoregulatory demands of the fish increase (Diep et al., 2015). Although the zebrafish kidneys have been well characterized, the distal excretory component of the urinary tract is not well studied and it is unclear whether a bladder and urethra exist or whether urine exits the body separately from the intestinal tract (Holden et al., 2013; Outtandy et al., 2019).

Here, we describe the zebrafish distal excretory urinary tract. We identify features similar to those observed in larger teleosts and mammals, including a urinary bladder that leads to a distinct urethra transporting urine externally. We also demonstrate intermittent micturition functionally *in vivo*.

## RESULTS

### Uroplakin genes in the zebrafish

As uroplakin expression is characteristic of human urothelium, we searched for conserved orthologues within the zebrafish genome. We identified three candidate genes: *uroplakin 1a* (ENSDARG00000021866.9), *uroplakin 2* (ENSDARG00000103164.2) and *uroplakin 3b* (ENSDARG00000092872.2). Protein alignment revealed structural homology and inter-species conservation (Figs S1-S3). Uroplakin 1a is a member of the tetraspanin superfamily of transmembrane proteins, which have four transmembrane helices (three clustered towards the N terminus and one at the C terminus). Uroplakin 2 and Uroplakin 3 have a single transmembrane domain towards the C terminus. Mammalian uroplakin 1a and uroplakin 1b interact with uroplakin 2 or uroplakin 3a to form two heterodimers that can subsequently exit the endoplasmic reticulum. The two components of this dimer (1a/1b and 2/3a) function together as a unit (Garcia-Espana et al., 2006).

### Uroplakin 1a mRNA expression in the zebrafish larval pronephros

**Fig. 1.**
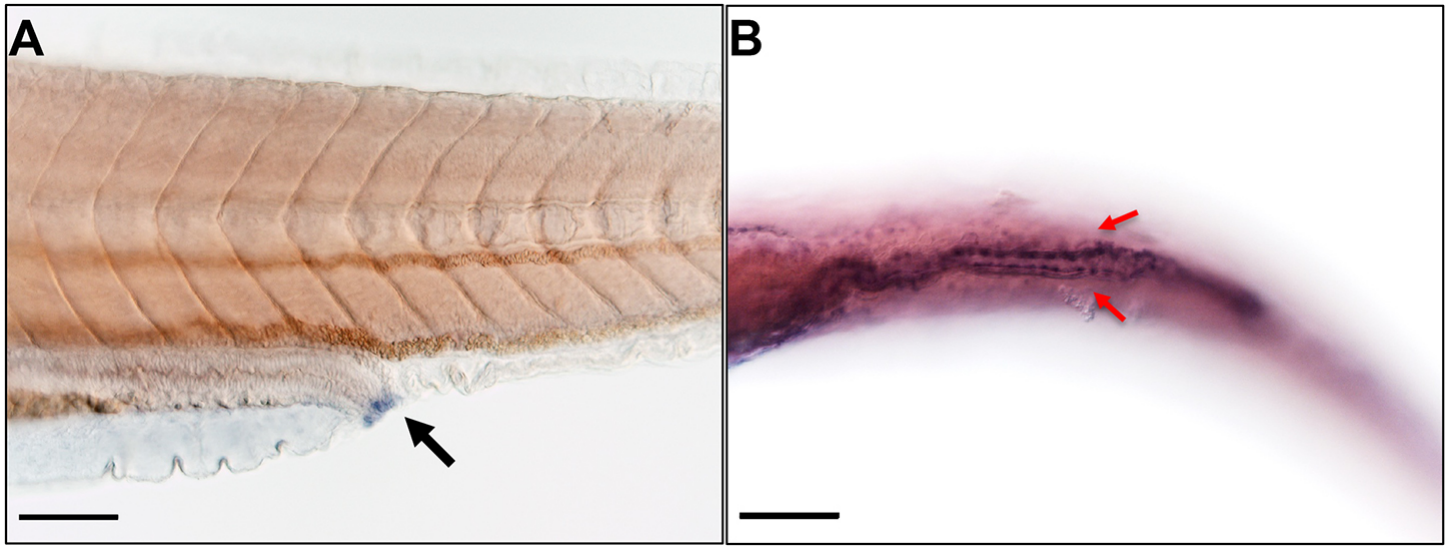
**Expression of *uroplakin 1a* in zebrafish larvae using *in situ* hybridization.** (A) *uroplakin 1a* expression in the cloaca (black arrow) at 96 h post fertilization (hpf) in lateral view. (B) *uroplakin 1a* expression in the two pronephric ducts (red arrows) as they drain from the kidney towards the cloaca at 120 hpf in ventral view. Scale bars: 50 μm.

Uroplakin 1a is the most highly conserved uroplakin and the only member present in zebrafish from the Uroplakin 1a/1b component of the dimer. *In situ* hybridization was performed to determine whether *uroplakin 1a*, a gene with high and specific expression in human urothelium, was expressed in the zebrafish pronephros (the precursor to the adult zebrafish urinary tract). We observed *uroplakin 1a* expression from around 96 hpf (hours post fertilization) in the zebrafish larval pronephros (Fig. 1, Fig. S4).

### Adult zebrafish demonstrate conserved anatomy of the excretory urinary tract

**Fig. 2.**
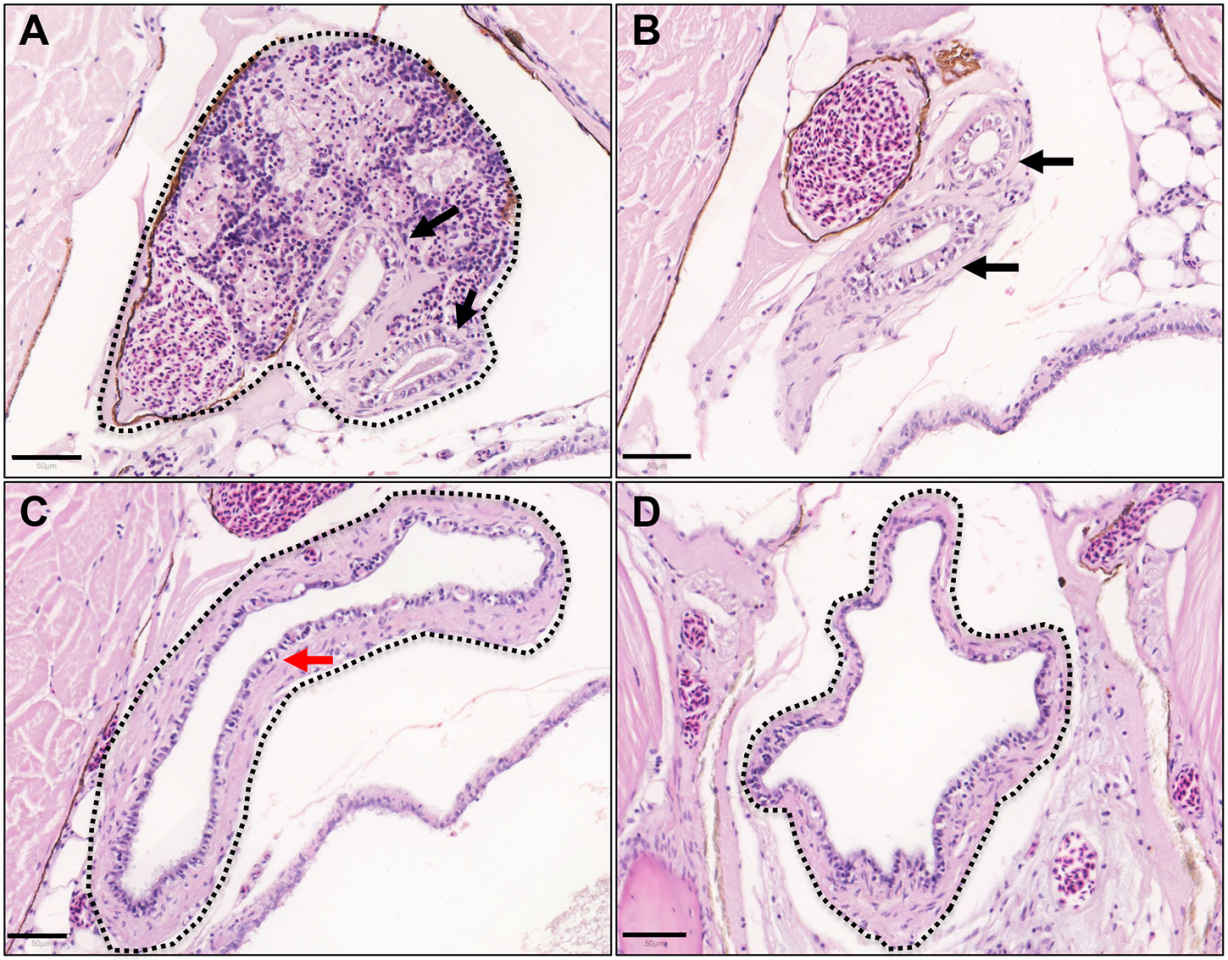
**Anatomy of adult zebrafish mesonephric ducts and urinary bladder.** Haematoxylin and Eosin stained adult zebrafish sections in transverse orientation from cranial to caudal. (A) Kidney (black outline) converges onto two mesonephric ducts (black arrows). (B) Two mesonephric ducts (black arrows) distal to the kidney in the retroperitoneum. (C) The two mesonephric ducts unite into a single urinary bladder lined with epithelial cells (red arrow). (D) Inferior aspect of the urinary bladder. Scale bars: 50 μm.

To explore any similarity between the anatomical structures of zebrafish and human excretory urinary tracts, we sectioned and stained adult zebrafish from the kidney to the cloacal region in transverse, coronal and sagittal orientations. Microscopy revealed two mesonephric ducts leaving the distal kidney, traveling inferiorly and caudally before fusing into a single collecting vesicle, with features of a urinary bladder (Fig. 2). This structure closely resembles the ureters and urinary bladder in humans. In contrast to human urothelium (which has multiple layers and different cell types), the zebrafish urinary bladder epithelium appeared to be one or two cells thick and all cells had a similar appearance (regardless of luminal proximity).

**Fig. 3.**
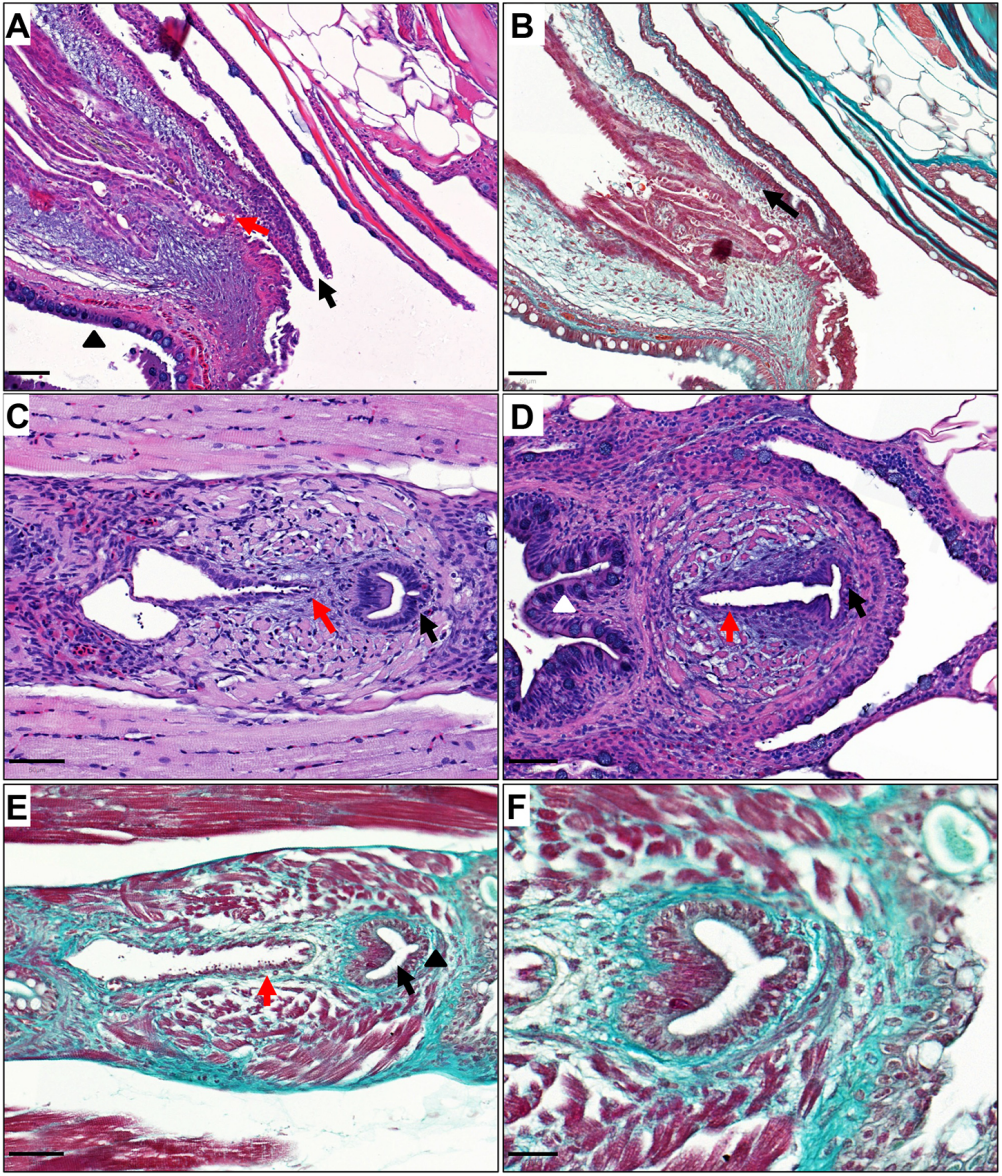
**Anatomy of adult zebrafish urethra.** (A) Haematoxylin and Eosin staining (sagittal orientation) of the adult female zebrafish lower urinary, intestinal and reproductive tracts, demonstrating a distinct and separate urethral orifice (black arrow), an oviduct (red arrow) and a rectum (black triangle). (B) Masson's trichrome staining demonstrating connective tissue (light green, black arrow) surrounding the female urethra. (C) Haematoxylin and Eosin staining (coronal orientation) of the adult male zebrafish lower urinary, intestinal and reproductive tracts, demonstrating a urethra (black arrow) and an ejaculatory duct (red arrow). (D) Haematoxylin and Eosin staining (coronal orientation) of the adult male zebrafish lower urinary, intestinal and reproductive tracts, demonstrating an ejaculatory duct (red arrow) opening into the urethra (black arrow) and a distinct separate rectum (white arrowhead). (E) Masson's trichrome stain of male adult lower urinary, intestinal and reproductive tracts (coronal orientation), demonstrating muscle fibres surrounding the ejaculatory duct (red arrow) and connective tissue (light green, black triangle) surrounding the urethra (black arrow). (F) Masson's trichrome staining of the male zebrafish urethra at higher magnification. Scale bars: 50 μm for A-E; 20 μm for F.

**Fig. 4.**
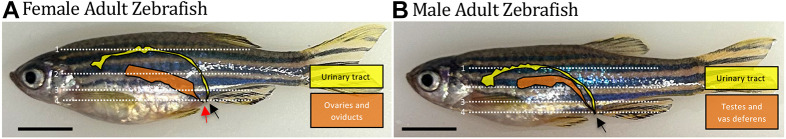
**Schematic of urinary tract and gonadal structures in adult zebrafish.** The urinary tract and oviducts in female adult zebrafish (A) exit via a separate urethral orifice (black arrow) and oviduct opening (red arrow), respectively. (B) The urinary tract and vas deferens in male adult zebrafish both exit via the urethral orifice (black arrow). Dotted lines illustrate the anatomical locations of key urinary tract structures in the coronal sectioning plane. Dotted line 1, coronal plane of section for the trunk of the kidney; dotted line 2, coronal plane of section for the ureter; dotted line 3, coronal plane of section for the urinary bladder; dotted line 4, coronal plane of section for the urethra. Scale bars: 0.5 cm.

Similarities between adult zebrafish and humans were also observed in the anatomy and morphology of the urinary tract structures that were distal and/or inferior to the urinary bladder. The urinary bladder in adult zebrafish, as for humans, leads to a urethra and urethral orifice that excretes urine (Fig. 3). Furthermore, Haematoxylin and Eosin, and Masson's trichrome staining demonstrated that the urethra in both males and female adult zebrafish is lined by an epithelial layer surrounded by connective tissue rich in collagen (Fig. 3). This histological characteristic is also present in the human urethra. In female adult zebrafish, the urethra and oviduct are separate tubes throughout their lengths (Fig. 3A,B and Fig. 4). However, in male adult zebrafish, the ejaculatory duct joins into the urethra, enabling ejaculation to occur via the urethra, which closely resembles ejaculation in male humans (Fig. 3C,D and Fig. 4).

**Fig. 5.**
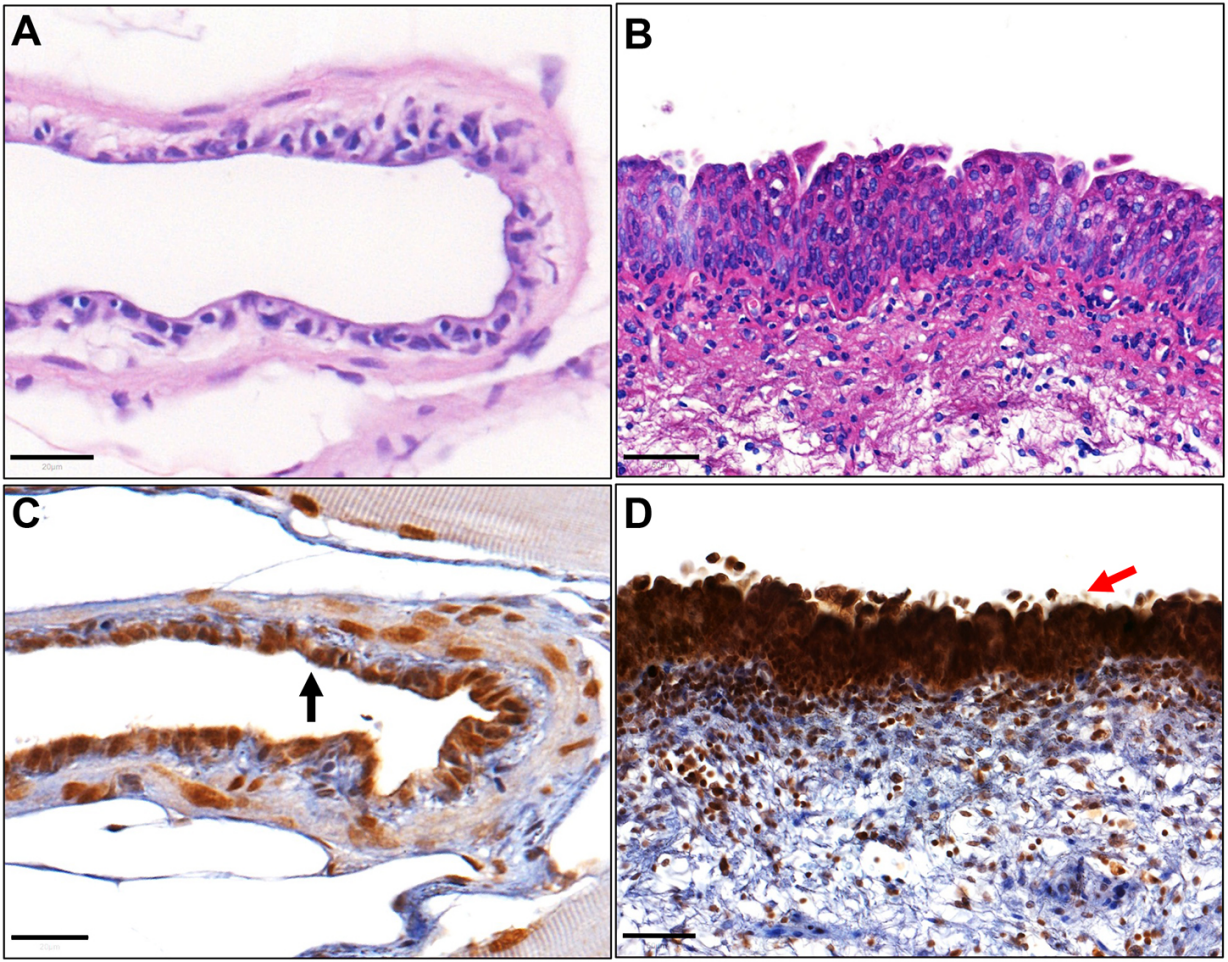
**Expression of GATA3 in zebrafish and human urinary bladder tissue sections using immunohistochemistry.** (A,B) Haematoxylin and Eosin staining of a coronal zebrafish urinary bladder section (A) and a human urinary bladder section for reference (B). (C) Nuclear expression of GATA3 in zebrafish urinary bladder urothelium (black arrow). (D) Nuclear expression of GATA3 in human urinary bladder urothelium (red arrow). Scale bars: 20 μm in A,C; 50 μm in B,D.

To understand protein conservation between the zebrafish and human excretory urinary tracts, we determined the expression of various proteins that are characteristic of human urothelium. GATA3 is a pioneer transcription factor and urothelial differentiation marker that is highly expressed in normal human urothelium (Miettinen et al., 2014). Immunohistochemistry revealed strong nuclear GATA3 expression in the zebrafish urothelium that matches human urothelial expression (Fig. 5, Fig. S5).

**Fig. 6.**
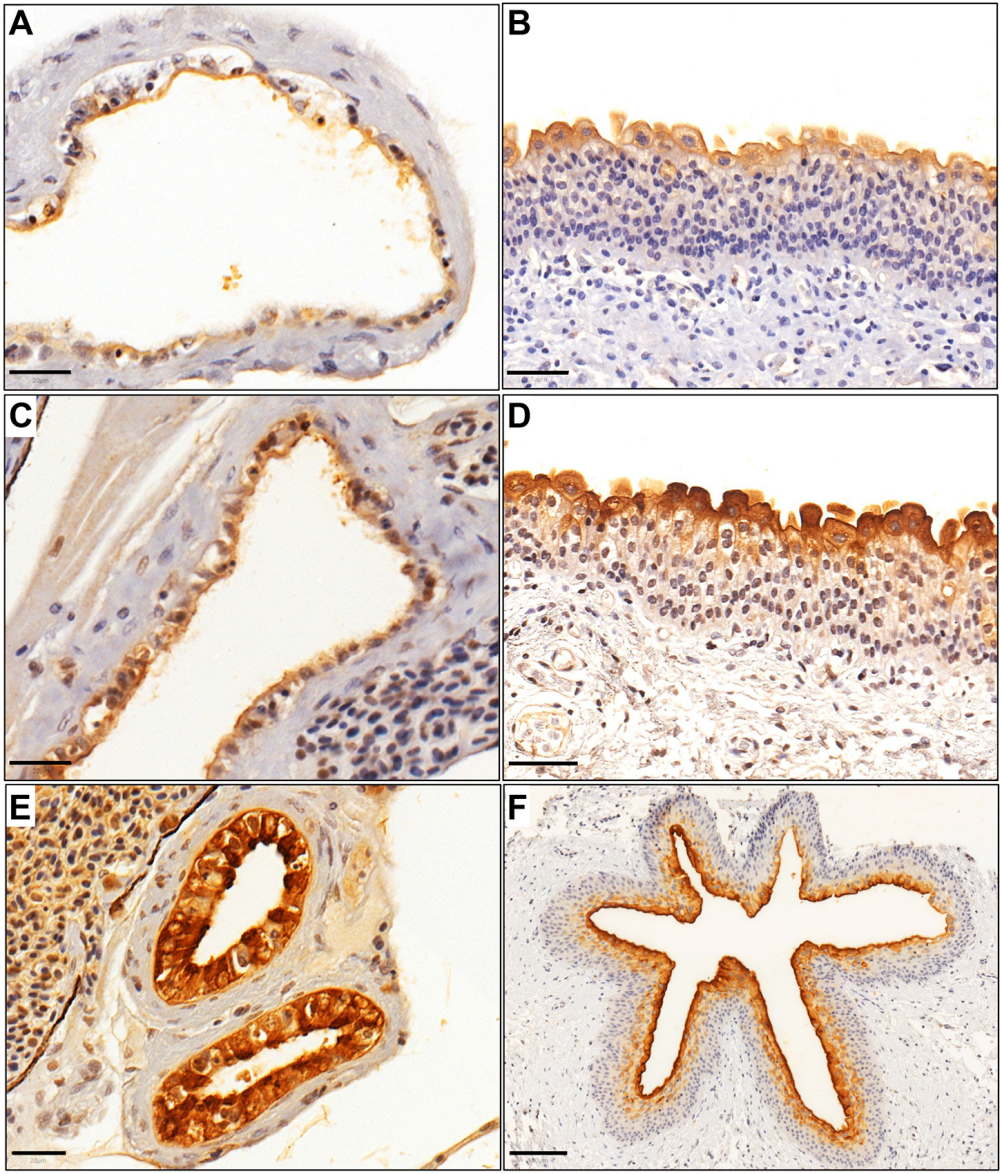
**Expression of uroplakins in zebrafish and human urinary bladder tissue sections using immunohistochemistry.** (A) Cytoplasmic and membranous expression of the urothelium-specific protein Uroplakin 1a (Upk1a) in zebrafish urinary bladder urothelium. (B) Cytoplasmic UPK1A expression in human urinary bladder urothelium. (C) Uroplakin 2 (Upk2) expression in a zebrafish urinary bladder. (D) UPK2 expression in a human urinary bladder. (E) Upk2 expression in zebrafish mesonephric duct epithelium. (F) UPK2 expression in human ureter urothelium. Scale bars: 20 μm for A,C,E; 50 μm in B,D; 100 μm in F.

Uroplakins are tetraspanin dimers that are characteristic of mammalian urothelium (Dalghi et al., 2020). Immunohistochemistry revealed cytoplasmic and membranous expression of Uroplakin 1a and Uroplakin 2 within the epithelium of the zebrafish urinary bladder and mesonephric duct, in a pattern similar to that seen in human urothelium (Fig. 6, Fig. S5).

**Fig. 7.**
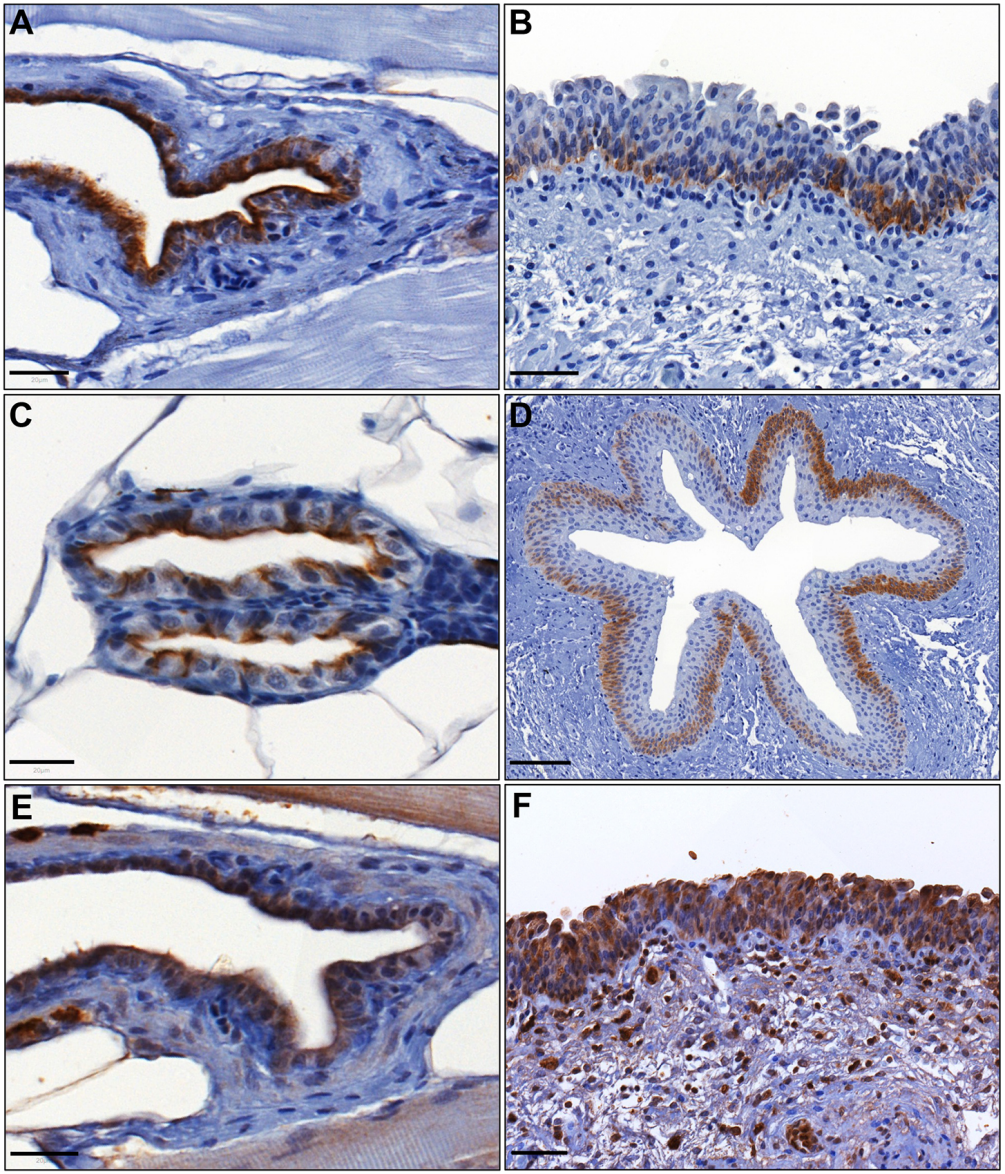
**Expression of urothelial basal cell layer markers in zebrafish and human urinary bladder tissue sections using immunohistochemistry.** (A) Cytoplasmic expression of Krt5 in zebrafish urinary bladder urothelium. (B) Cytoplasmic KRT5 in human urinary bladder urothelium. (C) Cytoplasmic Krt5 expression in zebrafish mesonephric duct epithelium. (D) Cytoplasmic KRT5 expression in human ureter urothelium. (E) Cytoplasmic CD44 expression in zebrafish urinary bladder urothelium. (F) Cytoplasmic CD44 expression in human urinary bladder urothelium. Scale bars: 20 μm for A,C,E; 50 μm for B,F; 100 μm for D.

Having demonstrated expression of proteins found in the superficial urothelial layer, we performed immunohistochemistry for a known marker of human basal urothelial layers, KRT5 (Volkel et al., 2022), and for the basal and stem cell marker CD44 (McKenney et al., 2001). Cytoplasmic KRT5 expression was confirmed in both the zebrafish urinary bladder and mesonephric duct epithelium. However, there was a difference between the expression of KRT5 between zebrafish and human urothelium: KRT5 was expressed throughout the zebrafish urinary epithelium but only in the basal cell layer of the human urothelium (Fig. 7A-D, Fig. S5). The basal and stem-like marker CD44 showed cytoplasmic expression in the zebrafish urinary bladder urothelium, in a similar manner to the human urinary bladder urothelium (Fig. 7E,F).

**Fig. 8.**
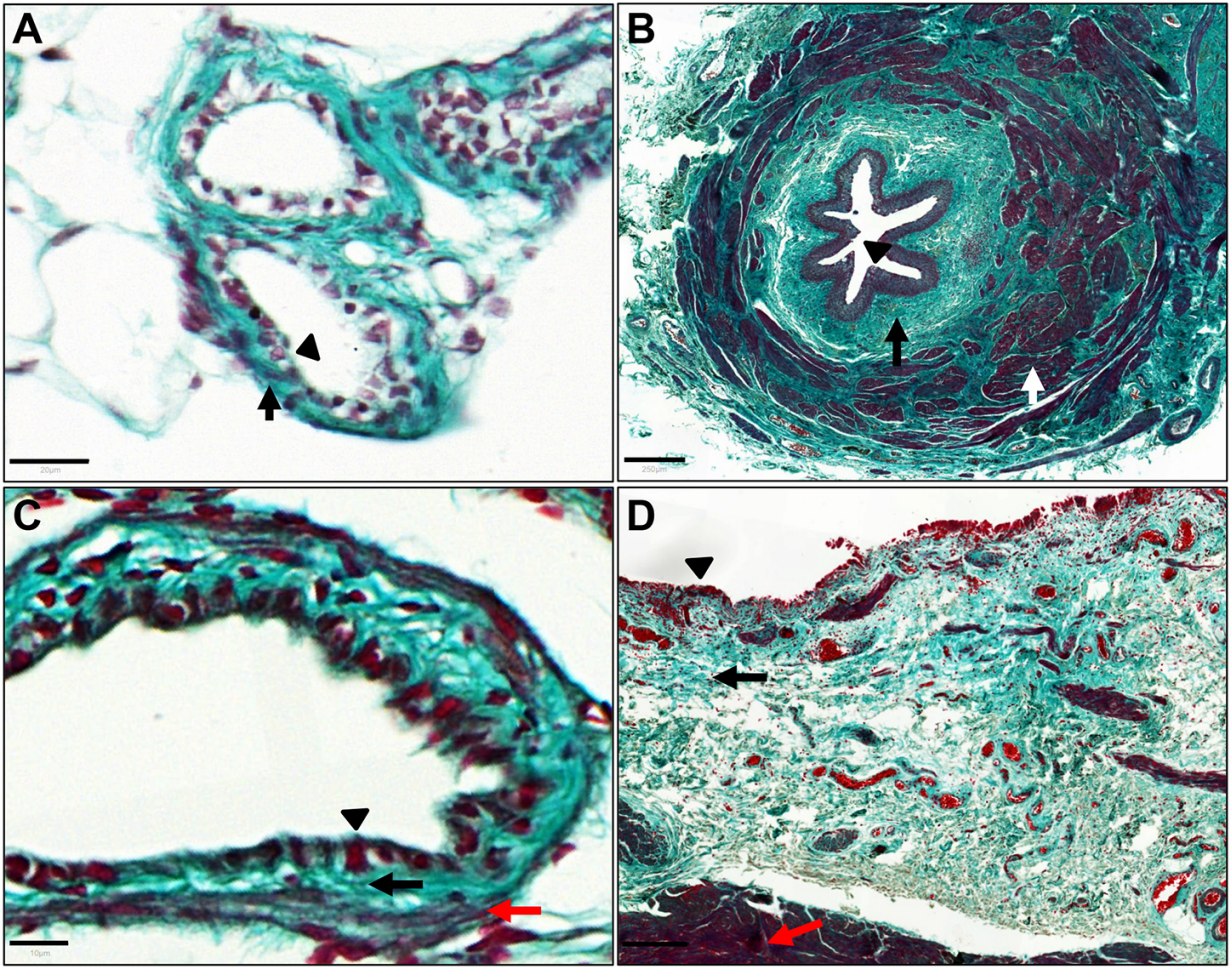
**Structure of zebrafish mesonephric duct and urinary bladder wall.** (A) Masson's trichrome staining of a zebrafish mesonephric duct section (coronal orientation) demonstrating a connective tissue layer (light green, black arrow) deeper than the epithelial layer (black triangle). (B) Masson's trichrome staining of a human ureter section, for comparison, demonstrating a connective tissue layer (light green, black arrow) deeper than the epithelial layer (black triangle) and muscle layer (white arrow). (C) Masson's trichrome staining of a zebrafish urinary bladder section (coronal orientation), demonstrating, deeper than the epithelial layer (black triangle), a connective tissue layer (light green, black arrow) and thin muscle fibres (red fibres, red arrow). (D) Masson's trichrome staining of human urinary bladder section demonstrating, deeper than the epithelial layer (black triangle), a connective tissue layer (light green, black arrow) and a muscle layer (red fibres, red arrow). Scale bars: 20 μm in A; 250 μm in B; 10 μm in C; 200 μm in D.

Masson's trichrome straining demonstrated that the wall of the zebrafish mesonephric ducts and urinary bladder have a connective tissue layer rich in collagen deeper than the epithelial layer. Similar to the zebrafish mesonephric duct, the human ureter also has a connective tissue layer deeper than the epithelial layer (Fig. 8A,B). However, the human ureter has a muscle layer deeper than the connective tissue (lamina propria) layer, which is not present in the zebrafish mesonephric duct. Masson's trichrome staining demonstrated the zebrafish urinary bladder wall has, deeper than the epithelial layer, a connective tissue layer and a deeper thin layer of muscle fibres. The structure of the human urinary bladder wall follows a similar pattern (Fig. 8C,D).

### *In vivo* analysis of storage and voiding function in adult zebrafish

**Fig. 9.**
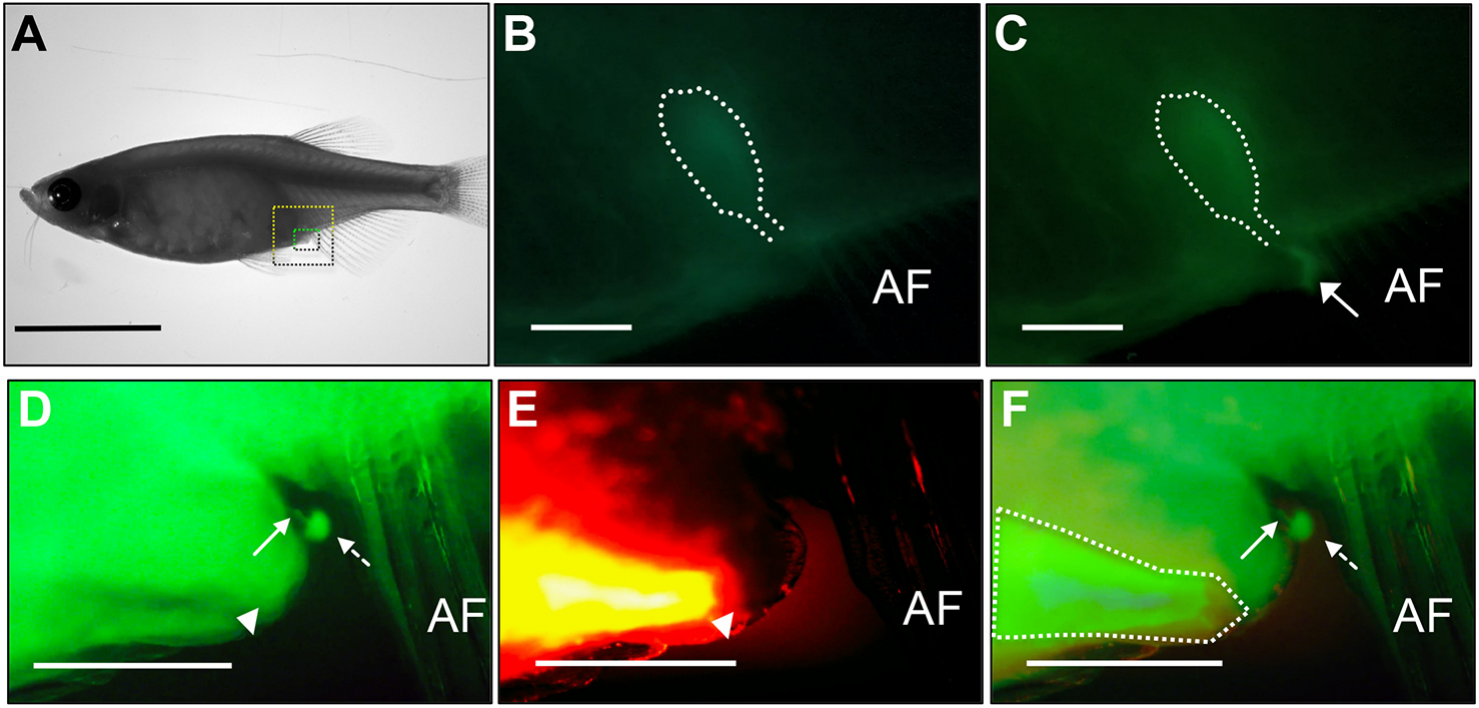
**Antegrade and retrograde urinary tracing studies in live adult zebrafish.** Alexa 488-conjugated dextran was injected into the pericardial space and imaged in the pelvic region. (A) Bright-field image of a zebrafish with areas shown in B,C highlighted in yellow and areas shown in D-F highlighted in green. (B,C) 10 min (B) and 13 min (C) post-injection. Urinary bladder structure is outlined in white, and the expulsion of urine is marked with a white arrow. (D) Epifluorescent imaging demonstrates urinary flow to a urethra (white arrow) posterior to the anal canal (white triangle). This urinary structure is unique to the anal canal and separately expels urine (white dotted arrow). (E) Retrograde counter-injection of dextran-conjugated Alexa 568 into the anal canal (white triangle) reveals the outline of the anorectum in orange. (F) Merged image identifies a urinary channel (white arrow) separate from the anal canal (outlined in white), and the expulsion of urine (white dotted arrow). The anal fin (AF) is indicated for reference. Scale bars: 1 cm in A; 300 μm in B,C; 5 mm in D-F.

To trace the course of urine in the excretory component of the zebrafish urinary tract, anterograde filling of the urinary system via pericardial injection and retrograde filling of the anal canal and rectum were performed with dextran-conjugated Alexa dyes on anaesthetized adult zebrafish (Fig. 9). Cannulation of the anal canal and rectum for the retrograde studies was performed carefully using a 1 µl syringe and ∼0.2 µl of dextran-conjugated Alexa 568 was injected. The pericardial space was injected with Alexa 488. The fish were imaged on a confocal microscope over 10-20 min. By ∼13 min after pericardial injection, urine (marked by green fluorescence) had accumulated in the urinary bladder region and micturition was demonstrated ventral to the urinary bladder (Fig. 9A-C). Intermittent micturition continued for several minutes and demonstrated periods of pausing (Movie 1). Excreted urine was regularly washed away using a pipette in order to continually visualize the micturition process. Confocal images of the region of urinary expulsion (marked by pericardially injected Alexa dye) demonstrated the urethral orifice as a tubular structure separate from the anal canal opening (Fig. 9D-F).

**Fig. 10.**
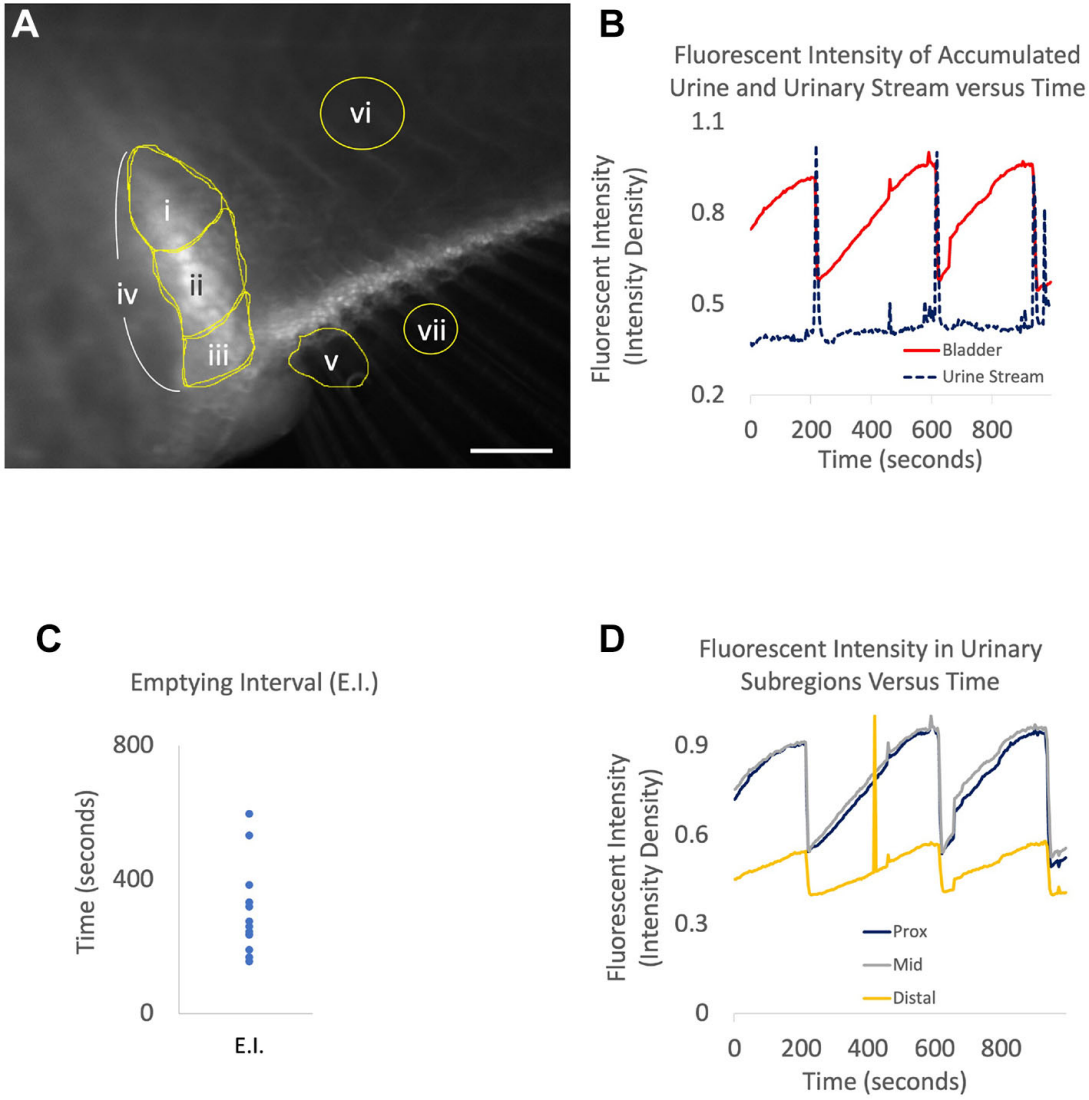
**Quantification of urinary excretion in zebrafish.** (A) After pericardial Alexa 488 injections, serial images were taken of zebrafish urinary structures. Scale bar: 0.4 mm. Regions of interest were identified as follows: i, proximal bladder; ii, middle bladder; iii, distal bladder; iv, total bladder; v, urinary stream. vi and vii were selected for normalization of body and urinary streams, respectively. (B) Plots of total bladder (iv) fluorescence intensity were plotted alongside urinary stream (v) intensity over time. (C) The distribution of bladder emptying intervals (E.I.) in seconds was plotted. (D) Bladder subregion fluorescent intensity plotted against time reveals that simultaneous contraction, and not sequential peristalsis, empties the full urinary bladder.

Although the injection studies demonstrated a bladder structure and separate orifice, urine accumulation in a non-partitioned imaging dish prevented satisfactory quantification of urinary function. We therefore designed an independent, two-chambered apparatus allowing for continuous exposure to tricaine anaesthesia in the gill region, combined with continuous flushing of the excreted urine at a stable rate, thus permitting quantification of voiding function. Movies were analysed to first identify the correlation of bladder emptying with urinary excretion (Movie 2). Fluorescent quantification of dye demonstrates that episodes of bladder emptying coincide with high volume voiding via the urethral meatus (Fig. 10A,B). Average bladder emptying interval (E.I.) was 302±37 s (s.e.m.) (*n*=4 animals, 13 E.I.s, Fig. 10C). The mechanism of emptying was further investigated by examining sequential segments of the bladder structure. Minor contractions were seen in the bladder during filling, but bladder emptying regularly occurred with a concerted contraction that involved simultaneous emptying of each segment of bladder (proximal, middle, and distal, Fig. 10D). Peristaltic emptying, where sequential segments propel urine along the bladder to excrete, was not present. In addition to well-correlated large voids, smaller or more frequent voids were also occasionally observed without significant emptying.

## DISCUSSION

Here, we have characterized the distal excretory component of the urinary tract in both larval and adult zebrafish, and demonstrate close homology in distal urinary tract anatomy and function between zebrafish and human. We have identified expression of *uroplakin 1a*, a characteristic urothelial gene, in the zebrafish embryo pronephros starting at 96 hpf, and have characterized key components of the adult zebrafish urinary excretory system (the urinary bladder arising from fusion of the two mesonephric ducts). We demonstrate that the zebrafish urinary bladder empties via a distinct urethra, and that, in anaesthetized adult zebrafish, urine accumulates in the bladder and is intermittently released.

Using whole-mount *in situ* hybridization, we detected the presence of *uroplakin 1a* expression in the zebrafish larval pronephric tubules and ducts at 96 hpf and 120 hpf. This is supported by the results of a previous study demonstrating expression of another uroplakin protein, Uroplakin 3b (Ukp3b), at the apical surface of the zebrafish pronephric tubules and ducts (Mitra et al., 2012). Morpholinos against *upk3b* led to embryos with reduced pronephric function believed to be due to defects in epithelial polarity and morphogenesis. The zebrafish pronephric epithelium and the human urothelium both play roles in urine excretion function, and are in direct contact with urine. Our findings suggest a structural homology between these two structures. Uroplakins are transmembrane proteins that form an asymmetric unit membrane (AUM) on the apical surface of the superficial layer of human urothelium (Dalghi et al., 2020). Multiple functions have been reported for uroplakins. Mouse knockout studies suggest that they play a role in the apical membrane permeability barrier function of urothelium (Hu et al., 2000, 2002). Abnormal uroplakins have also been linked to urinary tract malformations such as hydronephrosis and vesicoureteric reflux in mouse knockout and human genetic studies (Jenkins et al., 2005; Kong et al., 2004).

We demonstrate that zebrafish possess two mesonephric ducts arising from the kidneys and uniting into a urinary bladder. The zebrafish mesonephric ducts and urinary bladder are lined with an epithelium expressing proteins that are characteristic of human urothelium (suggesting structural similarity and evolutionary conservation). These are important findings because previously there have been conflicting reports regarding the presence of a urinary bladder in zebrafish (Baranowska Körberg et al., 2015; Holden et al., 2013; Outtandy et al., 2019). The presence of mesonephric ducts and a urinary bladder have been described in other teleost species (Curtis and Wood, 1991; Macrì et al., 2014). We did note two differences between the zebrafish and human urothelium. First, the zebrafish urinary bladder epithelium is one or two cell layers thick, in comparison with the multi-layered human urothelium. Second, human urothelium is composed of superficial (‘umbrella’), intermediate and basal cell layers, which are characterized by differential protein expression (Dalghi et al., 2020). The zebrafish urinary bladder epithelium expressed proteins characteristic of both superficial (uroplakins) and basal (Cytokeratin 5 and CD44) layers. This may reflect biological adaptation to differences in urinary composition, anatomical simplicity or that proteins characteristic of luminal and basal layers are different in zebrafish and humans.

This study also shows structural similarity in the lower urinary tracts distal to the urinary bladder in zebrafish and humans. We demonstrate that the zebrafish urinary bladder leads to a urethra and to a urethral orifice that is separate from the hindgut and surrounded by a deeper connective tissue layer rich in collagen. These structures have not previously been described in zebrafish. Furthermore, we show sex differences in urethral anatomy in zebrafish by demonstrating that the male zebrafish ejaculatory duct joins the urethra (similar to humans), whereas the female oviduct is a separate structure to the urethra throughout its course.

In anaesthetized adult zebrafish, we showed *in vivo* that urine is stored and intermittently released. Intermittent release of urine has been reported in another larger teleost species, the freshwater rainbow trout, in which urine is stored for 25-30 min (Curtis and Wood, 1991). Urine storage in the freshwater rainbow trout enables solute reabsorption (sodium and chloride ions) to take place, functioning as an adjunct to kidney function (Curtis and Wood, 1991). Although this is not considered to be a traditional function of the mammalian urinary bladder, recent data in a porcine model is supportive of urinary bladder reabsorption function in mammals (Manso et al., 2019). Urine storage in the freshwater rainbow trout has also been suggested as being potentially important for survival by avoiding the persistent release of olfactory signals to potential nearby predators (Curtis and Wood, 1991).

### Conclusions

The adult zebrafish distal urinary excretory system shares several features with its human counterpart. The zebrafish system has two mesonephric ducts leading to a urinary bladder lined by a uroplakin-expressing urothelium. The urinary bladder, in turn, leads to a urethra and urethral orifice. These findings present an opportunity to model human lower urinary tract diseases in zebrafish.

## MATERIALS AND METHODS

### Genome alignment

Genomic DNA and protein sequences for uroplakin genes were identified in humans (*Homo sapiens*), mouse (*Mus musculus*) and zebrafish by searching the NBCI genome and protein browser. Protein sequences were aligned using MUSCLE tool on Snapgene (Version 4.3.11).

### Zebrafish maintenance and strains

Zebrafish were raised and housed in the Bateson Centre at the University of Sheffield in aquaria approved by the UK Home Office and maintained according to standard protocols (maintained at 28.5°C) in accordance with the UK Animals (Scientific Procedures) Act 1986. Wild-type zebrafish were from stocks held at the Sheffield Biological Services Aquaria.

### Histology

To obtain zebrafish paraffin wax-embedded tissue sections, male and female wild-type adult zebrafish (3 months to 3 years old) were culled and subsequently fixed in 10% formalin or 4% paraformaldehyde for 3-4 days at 4°C or 2 days at room temperature. The samples were decalcified using 0.25 M EDTA (pH 8) at room temperature for 2-4 days and then immersed in 70% ethanol. The samples were dehydrated through ascending concentrations of ethanol (70% to 100%) and transferred to xylene, before infiltration and embedding in paraffin wax. A microtome was used to section wax-embedded fish at 3.5-5 μm. Fish samples were either oriented transversely, coronally or sagittally. Human bladder tissue was obtained with the ethical approval of the South Yorkshire Research Ethics Committee (REC reference number: 10/H1310/73). For Haematoxylin and Eosin staining, tissue sections were dewaxed with xylene and rehydrated in descending concentrations of ethanol to water before staining.

For Masson's trichrome staining, sections were dewaxed in xylene and rehydrated in descending concentrations of ethanol to water, and subsequently mordanted in Bouin's solution for 1 h at 56°C. The sections were then stained with Wiegert's Haematoxylin, Ponceau Fuschin and Light Green SF. Differentiation was performed using phosphomolybdic acid solution before Light Green SF staining. This staining was carried out on multiple sections from two adult zebrafish (coronal and sagittal orientations).

### Immunohistochemistry

Sections were dewaxed and rehydrated. Endogenous peroxidase was blocked with 3% hydrogen peroxide/methanol solution (one part 30% hydrogen peroxide to nine parts methanol) at room temperature. The Avidin and biotin blocking kit [Vector Laboratories (now called 2BScientific)] was used (for KRT5, GATA3 and CD44 markers) to block signal from endogenous biotin, biotin receptors and avidin-binding sites present in tissues. A 10% serum block was used to reduce non-specific binding (rabbit and goat sera). The following antigen retrieval methods were used: 0.01 M citrate buffer (heated in a microwave) and DAKO Target Retrieval Solution (S1699) (heated in an antigen retriever).

The following primary antibodies were used: anti-Uroplakin 1a (N-16) (goat polyclonal) (1:400 dilution for zebrafish tissue; 1:200 dilution for human tissue; Santa Cruz Biotechnology, sc-15170); anti-Uroplakin 2 (k-18) (goat polyclonal) (1:400 dilution for zebrafish tissue; 1:100-1:400 dilution for human tissue; Santa Cruz Biotechnology, sc-15179), anti-Cytokeratin 5/6 (mouse monoclonal) (1:100-1:200 dilution for zebrafish tissue; 1:100 dilution for human tissue; Dako, M7237), anti-GATA3 (rabbit) (1:400 dilution for zebrafish tissue, 1:100 dilution for human tissue (Sigma Aldrich, SAB2100898) and anti-CD44 (mouse) at 1:400 dilution for zebrafish tissue and 1:50 dilution for human tissue (Sigma Aldrich, SAB1405590). The following secondary antibodies were used: biotinylated rabbit anti-goat [1:200; Vector Laboratories (now 2BScientific), catalogue no.: BA-5000-1.5], biotinylated goat anti-rabbit [1:200; Vector Laboratories (now 2BScientific), catalogue no.: BA-1000-1.5) and biotinylated goat anti-mouse [1:200; Vector Laboratories (now 2BScientific), catalogue no.: BA-9200-1.5]. Vectastain Elite ABC kit, Peroxidase [Vector Laboratories (now 2BScientific)] and DAB (3,3′-diaminobenzidine) substrate kit, Peroxidase [Vector Laboratories (now 2BScientific)] were used for detection. Each immunohistochemical marker was performed on sections from one adult zebrafish.

### Whole-mount *in situ* hybridization

For digoxygenin-labelled RNA probe production, the probe for *uroplakin 1a* was synthesized from linearized plasmid DNA obtained from a plasmid vector containing the coding sequence. The coding sequence was contained in the p-express 1 plasmid. The Zebrafish Information Network (ZFIN) was searched for details regarding zebrafish *upk1a* cDNA (MGC: 136832, Accession number: GenBank: BC115285). A cDNA clone for *upk1a* was obtained from Source BioScience, Nottingham, UK. This *upk1a* cDNA clone was then inserted in the pExpress 1 vector between EcoRV (744) and NOT1 (735) restriction sites. For the antisense *uroplakin 1a* probe, the p-express 1 plasmid was linearized using the EcoRI restriction enzyme and the T7 RNA polymerase enzyme was used for transcription. For the sense *uroplakin 1a* probe, the p-express 1 plasmid was linearized using the NotI restriction enzyme and the SP6 RNA polymerase enzyme was used for transcription. Whole-mount *in situ* hybridization was performed using standard procedures (Westerfield, 2000).

### Anatomical tracing studies

Adult zebrafish were anaesthetized with tricaine solution. Dextran-conjugated Alexa dyes (Thermo-Fisher) were prepared in phosphate-buffered saline and had a concentration of 10-20 µg/µl. Alexa 488 conjugated to 10 K MW dextran was used. Using 1 µl syringes, the anal canal and rectum were carefully cannulated and injected with ∼0.2 µl Alexa 568 and the pericardial space injected with Alexa 488. Fish were imaged for 10-20 min and maintained under anaesthesia with flushes of tricaine solution. For urinary function studies, zebrafish were similarly anaesthetized in titrated tricaine and only intracardiac injection was performed. Zebrafish were placed in a partitioned custom-built physiology chamber to allow a range of anaesthetic and flushing flows. Images were obtained every 4 s. Fish were sacrificed in ice-water baths after imaging.

### Imaging and software

The *in situ* hybridization images were obtained using Leica Microsystems DFC 420 C1.8 microscope. Anatomical tracing studies were performed with a Zeiss Axiocam 208 on a fluorescent Leica stereoscope. Images were captured with Labscope software (Zeiss). *In vivo* urinary studies were performed using the Zeiss AxioZoom steromicroscope and Zen software. Images were then selected for regions of interest, as identified in Fig. 10A, and serially measured for fluorescence intensity in FIJI, which was plotted against time. Confocal images were obtained on an Andor Spinning disk confocal microscope. Movies clips were edited using iMovie software. The Panoramic 250 Flash III slide scanner (3DHISTECH) was used to image the histology slides. Histology images were processed using Qupath version 0.1.2 (Bankhead et al., 2017).

## Supplementary Material

10.1242/dmm.050110_sup1 Supplementary information
