# Supplementary material for: Analysis of the distal urinary tract in larval and adult zebrafish reveals homology to the human system
Source: Dis Model Mech. 2023 Jul 19;16(7):dmm050110. doi: 10.1242/dmm.050110 (PMC10387350; doi:10.1242/dmm.050110)
Supplement: Supplementary information [file dmm-16-050110-s1.pdf]

```
                   10        20        30        40        50        60        70
HUMAN      MASAAAAEAEKGSPVVVGLLVVG-NIIILLSGLSLFAETIWVTADQYRVYPLMGVSGKDDVFAGAWIAIF  69
MOUSE      -MASAATEGEKGSPVVVGLLVVG-NIIILLSGLALFAETVWVTADQYRVYPLMGVSGKDDVFAGAWIAIF  68
ZEBRAFISH  ----------MGAVTCLMVTVVGLNAIAAAAGLALSAVAIWVAVDGYKLYPISGVSGKDDIFAGAWIAIF  60

                   80        90       100       110       120       130       140
HUMAN      CGFSFFMVASFGVGAALCRRRSMVLTYLVLMLIVYIFECASCITSYTHRDYMVSNPSLITKQMLTFYSAD  139
MOUSE      CGFSFFVVASFGVGAALCRRRYMILTYLLLMLIVYIFECASCITSYTHRDYMVSNPSLITKQMLTYYSAD  138
ZEBRAFISH  TGFAFFLTCIFGIFAALKRSRALMLVYLIIMFIIFLFESASAITSATNRDYLVGNSNLVKKQMLQYYADS  130

                  150       160       170       180       190       200       210
HUMAN      TDQGQELTRLWDRVMIEQECCGTSGPMDWVNFTSAFRAATPEVVFPWPPLCCRR-TGNFIPLNEEGCRLG  208
MOUSE      TDQGQELTRLWDRIMIEQECCGTSGPMDWVNYTSAFRAATPEVVFPWPPLCCRR-TGNFIPINEDGCRVG  207
ZEBRAFISH  STQGQQITMTWNNVMTQVQCCGADGPTDWIQYNSTYRQLFGAASL-WPLGCCKRQSSNFEVVDPIGCKAG  199

                  220       230       240       250       260
HUMAN      HMDYLFTKGCFEHIGHAIDSYTWGISWFGFAILMWTLPVMLIAMYFYTML-  258
MOUSE      HMDYLFTKGCFEHIGHAIDSYTWGISWFGFAILMWTLPVMLIAMYFYTTL-  257
ZEBRAFISH  VTSSMFTQGCFQYIESVLSRYTWAVSWYGFSVLMLVFFTLVIAMIYYTQLP  250
```

Fig. S1. Alignment of Human, Mouse and Zebrafish Uroplakin 1a Amino Acid Sequences. Muscle Tool used for alignment. Human sequence used as reference. Alignment highlighted in green.

Disease Models & Mechanisms • Supplementary information

```
                    10        20        30        40        50        60        70
HUMAN      MAPLLPIRTLPLILILLALLSPGAADFNISSLSGLLSPALTESLLVALPPCHLTGGNATLMVRRANDSKV  70
MOUSE      MASTLPVQTLPLILILLAVLAPGTADFNISSLSGLLSPALTESLLIALPPCHLTGGNATLMVRRANDSKV  70
ZEBRAFISH  -----------------------------------------------MPDCSIYGNQSVLLLYTEAPTNL  23

                    80        90       100       110       120       130       140
HUMAN      VTS-SFVVPPCRGRRELVSVVDSGAGFTVTRLSAYQVTNLVPGTKFYISYLVKKGTATESSREIPMSTLP  139
MOUSE      VKS-DFVVPPCRGRRELVSVVDSGSGYTVTRLSAYQVTNLTPGTKYYISYRVQKGTSTESSPETPMSTLP  139
ZEBRAFISH  NNTVNFTVQPCP-----------------VSQSWYLLGNLKNGTTYSMSYKI----GNDTSSVLTNTTTN  72

                   150       160       170       180
HUMAN      RRNMESIGLGM-ARTGGMVVITVLLSVAMFLLVLGFIIALALGSRK  184
MOUSE      RKNMESIGLGM-ARTGGMVVITVLLSVAMFLLVVGLIVALHWDARK  184
ZEBRAFISH  VNDYQQIDTGLRARSGAMVVITVILSLAMVFLLVGIILVFFFFSG-  117
```

Fig. S2. Alignment of Human, Mouse and Zebrafish Uroplakin 2 Amino Acid Sequences. Muscle Tool used for alignment. Human sequence used as reference. Alignment highlighted in green.

Disease Models & Mechanisms • Supplementary information

```
HUMAN      -----------MPPLWALLALGCLRFGSAVNLQPQLASVTFATNNPTLTTVALEKPLCMFDSKEA-----  54
MOUSE      -----------MLLLWALLALGCLRCGWTVNLQPQLASVTFATNNPTLTTVALEKPLCMFDSSEP-----  54
ZEBRAFISH  MNTHNAIRLVSLLSIWMLAAQGQI-------FQPQLAPANF-LGRITSNTVILQQPYCVFTQTCPGCEIW  62

HUMAN      -----LTGTHEVYLYVLVDSAISRNASVQDSTNTPLGSTFLQTEGGRTGPYKAVAFDLIPCSDLPSLDAI  119
MOUSE      -----LSGSYEVYLYAMVDSAMSRNVSVQDSAGVPLSTTFRQTQGGRSGPYKAAAFDLTPCGDLPSLDAV  119
ZEBRAFISH  LVAALSTGTGNFNALVNISSPISLSVSPYPTAFLPSSAQFFLT---RVGPLANF-----PCNTAPAF---  121

HUMAN      GDVSKASQILNAYLVRVGANGTCLWDPNFQGL-CNAPLSAATEYRFKYVLVNMSTGLVEDQTLWSDPIRT  188
MOUSE      GDVTQASEILNAYLVRVGNNGTCFWDPNFQGL-CNPPLTAATEYRFKYVLVNMSTGLVQDQTLWSDPIWT  188
ZEBRAFISH  -----------PYFT-VGADGIC------TGINCNGVLPVGSIVSFRYLLIDPSNYTVVNMTNWGGPFNL  173

HUMAN      NQLTPYSTIDTWPGRRSGGMIVITSILG-SLPFFLLVGFAGAIALSLVDMGSSDGETT----------HD  247
MOUSE      NRPIPYSAIDTWPGRRSGGMIVITSILG-SLPFFLLVGFAGAIILSFVDMGSSDGEMT----------HD  247
ZEBRAFISH  TTLLSYQTINDGLSARSGAMVVITTLLCVAVALLLLVFF---IMLCVSCCGKKDGKTVTVMSSIRIPRYD  240

HUMAN      SQITQEAV-PKSLGASESSYTSVNRGPPLDRAEVYSSKLQD  287
MOUSE      SQITQEAV-PKTLGTSEPSYSSVNRGPPLDRAEVFSSKLQD  287
ZEBRAFISH  THNLKEHVHPYDNQAYEPDAKNYSRSQTLPKSPVRK-----  276
```

Fig. S3. Alignment of Human, Mouse and Zebrafish Uroplakin 3b Amino Acid Sequence. Muscle Tool used for alignment. Human sequence used as reference. Alignment highlighted in green.

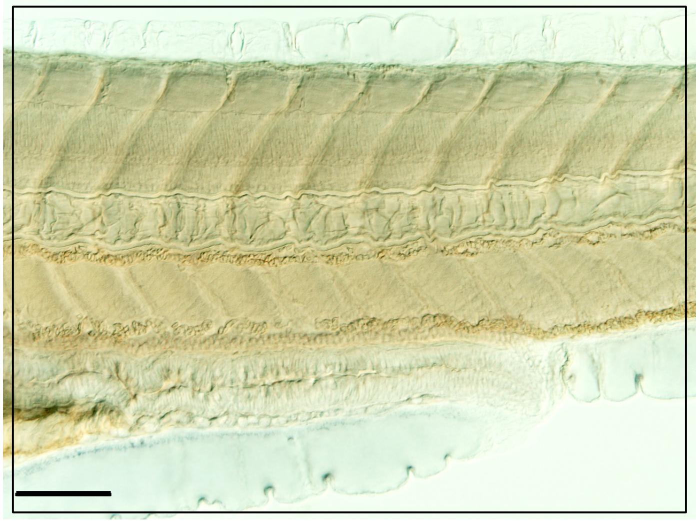

Fig. S4. Sense Control for *Uroplakin 1a In Situ* Hybridisation in Zebrafish larvae at 96 hpf. Scale bar= 50µm.

Disease Models & Mechanisms • Supplementary information

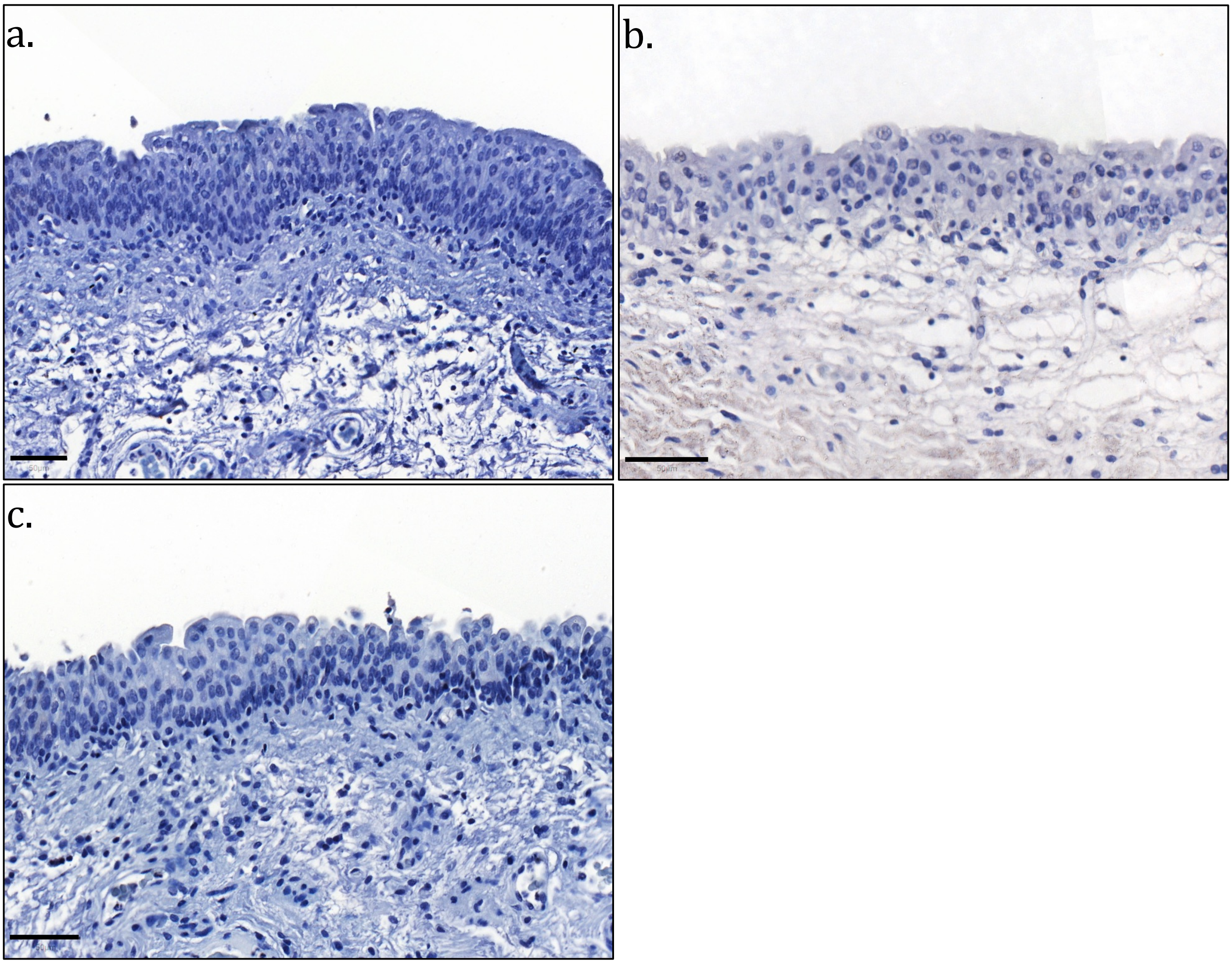

Fig. S5. Negative (No Primary Antibody) Controls for Immunohistochemistry. A. No primary antibody control for biotinylated goat anti-rabbit secondary antibody, **B.** No primary antibody control for biotinylated rabbit anti-goat secondary antibody. **C.** No primary antibody control for biotinylated goat anti-mouse secondary antibody. Scale bars= 50µm.

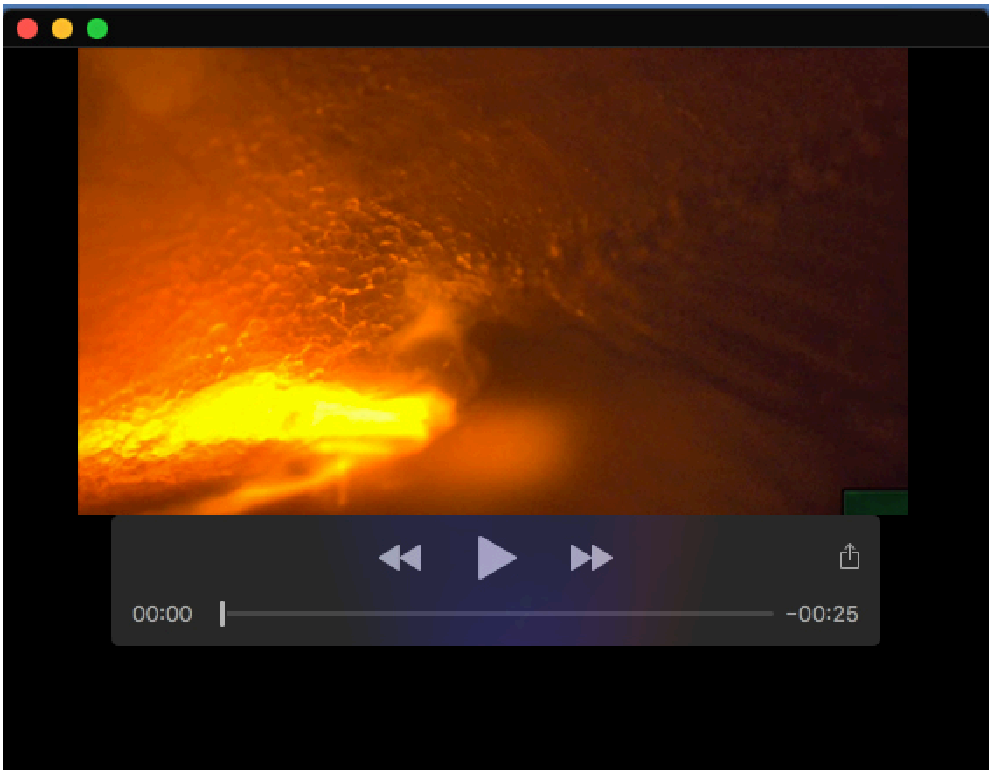

Movie 1. Micturition in Zebrafish. Pericardial injections were performed in adult fish as described. Counter injection into the cloacal channel was performed with dextran-conjugated Alexa 568. Separate channels were obtained by alternating prisms. Within 10 minutes, urine was expelled from the dorsal urethral meatus. Excreted urine was regularly washed away using a pipette in order to continually visualize the micturition process. Timescale is approximately 16x acquired speed. Images have same size scale as images in Figure 9d-f.

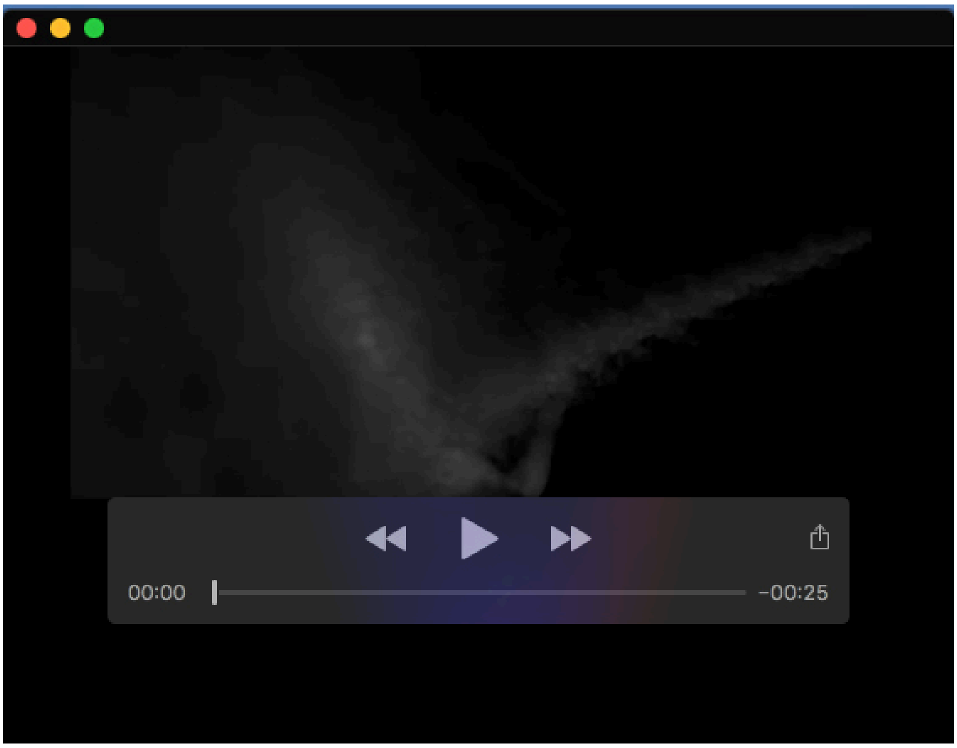

Movie 2. Bladder emptying with voiding activity. Pericardial injections were performed as described. Gentle flow of tank water removed excreted dye that was accumulated. Serial images were taken every 4 seconds. Movie is run at 32x speed. Scale bar=1mm.

Disease Models & Mechanisms • Supplementary information
